# A Model for Promoting Occupational Safety and Health in Taiwan’s Hospitals: An Integrative Approach

**DOI:** 10.3390/ijerph16050882

**Published:** 2019-03-11

**Authors:** Hui-Ting Huang, Chung-Hung Tsai, Chia-Fen Wang

**Affiliations:** 1 Taiwan Adventist Hospital, Taipei 10556, Taiwan; gih@tahsda.org.tw (H.-T.H.); 116426@tahsda.org.tw (C.-F.W.); 2 Department of Health Administration, Tzu Chi University of Science and Technology, Hualien 970, Taiwan

**Keywords:** perceived organizational support, safety climate, social influence, shared decision making

## Abstract

Advocating for improving workplace safety and health has gained substantial support in recent years. The medical industry is a high-risk industry and receives considerable public attention. This study used an integrative approach as a starting point and combined the contextual factors of an organization: perceived organizational support, safety climate, social influence, and shared decision making. Subsequently, the effects of these factors on preventive action and safety satisfaction were investigated. This study surveyed employees of two hospitals, one in Northern Taiwan and one in Eastern Taiwan, collecting valid data from 468 respondents. Structural equation modeling (SEM) was used to verify our research framework. The finding indicates that (1) All hypotheses proposed in this study were supported. (2) The overall goodness of fit of the model was excellent, and the explained variance of the outcome variables was high. (3) Safety climate had the strongest total effects on preventive action and safety satisfaction simultaneously, whereas preventive action had the strongest direct effect on safety satisfaction. The objective of this study was to obtain empirical conclusions and make suggestions for academic theory and clinical practice. The findings may serve as a reference for future research and for scholars and practitioners, enabling the creation of healthy workplaces and, thus, a brighter future.

## 1. Introduction

The latest statistics from the World Health Organization (WHO) reveal that approximately 122 million people die each year because of noninfectious diseases. These people are mostly from developing countries and of working age (15–64 years). Work-related health problems cause an economic loss of approximately 4% to 6% of the gross domestic product in most countries [1]. Therefore, occupational safety and health (OSH) should not be ignored in modern society. This is especially true for Taiwan, which is currently in an era of high competition. How attention can be paid to both OSH and the overall wellbeing of workers while transforming and developing Taiwan’s economy is a crucial topic for its government agencies and business enterprises. 

The starting point of this study was OSH. First, we investigated the concept of a safety climate. A safety climate is a particular form of organizational climate. Essentially, it refers to employees’ perceptions of safety-related policies, procedures, practices, and rewards in the workplace [2,3]. Zohar proposed this concept more than 30 years ago, but scholars have different definitions and have not reached a consensus on its dimensions [4]. In recent years, increasingly more scholars have reported that safety climate is a key antecedent of safety performance and that its importance is self-evident [5,6,7,8,9]. 

On an interpersonal influence level, the workplace is not only a physical environment; it also includes the social environment in which employees’ behaviors are governed by special norms [10]. The administration process of implementing OSH-related law and regulations, operations, and performance assessment requires input from employees. Therefore, social influences can predict the establishment of a friendly and high-quality working environment and contribute to its contextualization and socialization. 

The perspective of patient-centered care and patient participation is currently popular in the medical industry. Therefore, physician–patient relationships have also become cooperative. For these reasons, the shared decision-making (SDM) model has been proposed [11,12]. Several studies have verified that shared decision-making has a positive effect on medical outcomes, health care quality, and work satisfaction. Therefore, this study also involved shared decision making into the research framework.

An environment with excellent occupational safety cannot be created within only a short period of time. Business executives and managers must establish policies and allocate resources to support innovative and transformative thoughts and practices. Additionally, they should incorporate these new thoughts and practices into policy implementation, thereby forming social norms and a safety climate integrated into daily routines; this is the meaning of organizational support [13]. 

Based on the aforementioned perspectives, this study used two hospitals in Northern and Eastern Taiwan as study institutions and targeted all employees in these hospitals as the sample population. We investigated the effects of these aforementioned critical factors on OSH-related preventive action and safety satisfaction. The objective of this study was to clarify, determine, and verify the interactions between antecedence, mediation, and outcome variables. Finally, we aimed to serve as a reference to researchers in related fields, hospital executives, and work unit managers. 

### 1.1. Occupational Safety and Health

Occupational safety and health (OSH) is generally defined as “the science of anticipation, recognition, evaluation and control of hazards arising in or from the workplace that could impair the health and well-being of workers, taking into account the possible impact on the surrounding communities and the general environment” [14]. Amponsah-Tawiah suggested that OSH relates to not only employees’ overall wellbeing at work but also their overall physiological, psychological, and socially psychological safety and wellbeing [15]. The WHO uses the term “healthy workplace” to describe OSH and has defined it as a place in which improvements are continually made to ensure the safety, health, and wellbeing of workers [16]. 

Hospitals are currently the center of attention because of advancements in medical technology and the advent of aging societies. Much attention has also been paid to OSH issues. Preventing occupational injuries is of great importance to providing higher-quality patient service, improving the morale of medical teams, and increasing employee productivity. Personnel in hospitals are providers of medical services, and only medical personnel in good health can provide high-quality medical services, ensure safety, and improve patient health. Therefore, investigating the factors that influence the OSH of employees and strengthening the measures for preventing occupational injuries should be crucial sustainable management topics that require immediate action. 

### 1.2. Safety Climate

The concept of safety climate was first proposed by Zohar, who considered safety climate to be a specific form of an organizational climate. Safety climate refers to workers’ perceptions of how employers value safety of their workplace environment [4,9]. Zohar also developed dimensions for measuring safety climate: importance of safety training programs, management attitudes toward safety, effects of safe conduct on promotion, level of risk at workplace, effects of required work pace on safety, status of safety officer, effects of safe conduct on social status, and the status of safety committee [4].

Brown and Holmes simplified the measurement scale proposed by Zohar, proposing three dimensions of safety climate: management concern, management activity, and risk perception [17]. Dedobbeleer and Beland proposed two dimensions of perceived safety climate: management commitment and workers involvement [18]. Milijic, Mihajlovic, Strbac, and Zivkovic proposed seven dimensions of safety climate: safety awareness and competence, safety communication, organizational environment, management support, risk judgment and management reaction, safety precautions and accident prevention, and safety training [19]. 

Numerous studies have suggested that safety climate affects several crucial outcomes including performance of safety-related work practices, the success of safety-related behavior, accident frequency, and injury incidence. Griffin and Neal found that work safety climate directly influenced safety motivation, which indirectly influenced safety behavior [5]. Wilson et al. showed that the organizational climate indirectly affected work satisfaction through work design [20]. Tang et al. found that the safety climate indirectly affected the wellbeing of teachers through OSH-related knowledge and behavior (preventive action) [8]. Kearney et al. discovered a significant correlation between the safety climate and personal protective equipment usage behavior (preventive action) [9]. In terms of medical environments, Hofmann and Mark showed that the safety climate affected the satisfaction of nursing staff [6]. Cheah et al. discovered that the OSH management practices (preventive action) of hospitals had positive effects on safety satisfaction and safety feedback among nursing staff [7]. 

On the basis of these above discussions, we propose the following hypotheses:

**H1**: *Safety climate positively affects preventive action.*

**H2**: *Safety climate positively affects safety satisfaction.*

**H3**: *Preventive action positively affect* *s safety satisfaction.*

### 1.3. Social Influence

The subjective norm is a determining factor that influences behavioral intention in the theory of reasoned action (TRA) [21]. Consistent with TRA, which was a key theoretical underpinning for the original development of the technology acceptance model (TAM), Venkatesh and Davis [22] tap into social influence via subjective norm. The subjective norm was defined as an individual’s perception that most people who are important to them think they should or should not perform the behavior in question. According to the TRA, whether a person engages in a specific behavior is determined by their behavioral intention to engage in this behavior. Behavior intention is determined by personal attitude and subjective norms regarding the behavior. For example, if employees do not believe that managers and colleagues are concerned about OSH, they are extremely unlikely to believe that safety is important. 

Among the empirical studies on subjective norms that influence safety behavior, Fogarty and Shaw found that group norms are direct and indirect predictors of unsafe behaviors [23]. Javadi et al. discovered that normative beliefs and subjective norms were the factors that most strongly influenced the safety behavior of nursing staff [24]. Avci and Yayli found that the safety norms directly and indirectly influenced safety behavior [25]. Moreover, Abdullah et al. found that safety behavior was significantly affected by the attitudes and subjective norms of employees [26]. 

In terms of the effect subjective norms have on safety satisfaction, Lee, An, and Noh demonstrated that group norms (the social influence of managers and colleagues) affect work satisfaction [27]. Sardzôska and Tang showed a strong correlation between work environment (subjective norm) and work satisfaction [28]. Therefore, social influence has a positive effect on safety satisfaction. 

On the basis of these above discussions, we proposed the following hypotheses:

**H4**: *Social influence positively affects preventive action.*

**H5**: *Social influence positively affects safety satisfaction.*

### 1.4. Shared Decision Making

The provision of comprehensive service and proactive cooperation with patients, families, care providers, and communities is currently the focus of medical institutions, aiming to form a cooperative atmosphere. Moreover, the doctor–patient relationship has changed from dyadic and paternalistic to cooperative. The shared decision-making (SDM) model is emerging for these above reasons [11,12]. SDM is defined as a partnership relationship between health care providers and patients [29]. SDM is a process in which the patient and providers consider outcome probabilities and patients’ preferences and reach a health care decision based on mutual agreement [30]. SDM involves incorporating patients’ perspectives and values into clinicians’ treatment decisions. During SDM, doctors and patients make a decision together and consider the evidence for different treatment options; patients are thus encouraged to consider treatment plan options and communicate their preferences to the doctor [31]. In this process, factors such as empirical evidence regarding health care options, the knowledge and experience of the health care provider, and the values and preferences of the patient must be considered [32]. Overall, SDM coordinates each team member’s perspective leading to a decision obtained through mutual agreement [33]. 

Several studies have suggested that the shared decision-making model has a positive influence on medical results, OSH, and employee satisfaction. One study of an intensive care unit suggested that SDM was inversely correlated with the adverse event rate [34]. Reader, Flin, and Lauche [35] discovered that a poor-quality decision-making process could result in serious accidents. Légaré et al. [36] also proposed that SDM can improve the health care process. Murray et al. [37] indicated that participation in SDM enabled nurses to better control practical operations and gave them higher job satisfaction. 

In terms of the outcomes of SDM, Muller [38] showed that participatory management not only stimulated personal, professional, and organizational growth but also had positive effects on health care results. Saha and Kumar [39] also verified that participatory decision making had a positive effect on work satisfaction. Similarly, Dunn et al. [40] reported that SDM was strongly correlated with satisfaction with the decision-making process and the decisions themselves.

On the basis of these above discussions, we proposed the following hypotheses:

**H6**: *Shared decision making positively affects preventive action.*

**H7**: *Shared decision making positively affects safety satisfaction.*

### 1.5. Perceived Organizational Support

The perceived organization support (POS) theory uses the perspective of social exchange theory to explain the relationship between employees and organizations. Social exchange theory proposed that employment is a trade of effort and loyalty for tangible benefits and social rewards. Viewing the employee–employer relationship from a reciprocity norm perspective, any reward received by one party is reciprocal, and thus both parties benefit. POS refers to employees developing global beliefs concerning the extent to which the organization values their contributions and cares about their well-being. The favorable or unfavorable treatment that an employee receives is an indication that their organization favors or disfavors them. Moreover, if employees believe that the reward they receive is discretionary rather than due to an external restriction on the organization, their POS is stronger [13]. Voluntary organizational support is highly valued by employees because it indicates that the organization has genuine respect for its employees and honors their contributions [41]. 

Studies have suggested that when employees perceive their manager to show support and concern for their wellbeing, they have higher work satisfaction and more pro-social behaviors. These pro-social behaviors include employees’ safety behaviors [42]. In their empirical study, Hofmann and Morgeson [43] suggested that POS affects safety communication and thus safety commitment and accident occurrence. A later study by Hofmann, Morgeson, and Gerras [44] revealed that POS resulted in more instances of safety-related organizational citizenship behavior. Additionally, Gyekye and Salminen [42] suggested that workers with positive POS help create a positive workplace safety climate. 

In terms of the effect of POS on social influence, Rhoades and Eisenberger [13] observed that POS contributes to employees’ sense of purpose and meaning, thereby enhancing organizational commitment. Parsons and Shils [45] indicated that organizational commitment promotes committing toward social roles or statuses, social relations, norms, values, and beliefs. According to the aforementioned viewpoints of scholars, POS promotes the social influence within organizations. 

Numerous studies have suggested that POS has a positive effect on SDM. American Institutes for Research (AIR) [12] reported that two simultaneous approaches support SDM: providing information and support, and implementing financial incentives. Baggs et al. [46] indicated that the success of implementation of a decision-making model is determined by the leading physicians’ willingness to listen, the degree of SDM, and the supportive cooperation structure, which all promote coordination. Respecting and trusting other professionals and being willing to consider different viewpoints is crucial to successful SDM [47]. Additionally, open communication and participatory discussion abilities are essential to effective SDM [40]. Overall, proactive organizational support has positive effects on the success of promoting SDM. 

On the basis of these above discussions, we proposed the following hypotheses:

**H8**: *Perceived organizational support positively affects safety climate.*

**H9**: *Perceived organizational support positively affects social influence.*

**H10**: *Perceived organizational support positively affects shared decision making.*

**Figure 1:**
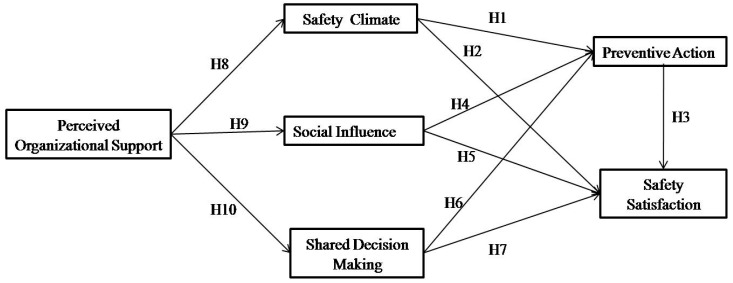
The proposed research model.

Based on the above 10 hypotheses, the proposed research model is illustrated in Figure 1.

## 2. Materials and Methods

### 2.1. Data Samples

This study adopted convenience sampling techniques to collect data. This study surveyed employees of two hospitals in Northern and Eastern Taiwan, and the questionnaire response period was six months (August 2017 to January 2018). The participation of surveyed employees in this study was all voluntary. These two hospitals’ managers helped to collect questionnaires. A total of 540 survey questionnaires were distributed, and 495 questionnaires were received. These received responses were excluded for (1) respondents that provided the same response for all items, (2) incomplete questionnaires, and (3) questionnaires with incomplete demographic information. Finally, we obtained 468 valid questionnaires, a valid response rate of 86.7%.

### 2.2. Measures

The items of the survey questionnaire were modified from previous studies. Each questionnaire item used a five-point Likert scale to measure constructs, ranging from 1 (strongly disagree) to 5 (strongly agree). It was also composed of several questions about demographic characteristics of participation, such as gender, age, marital status, educational level, department, and professional experience. Perceived organization support (POS) was measured using four items modified from DeConinck and Johnson [48]. Safety climate was measured using a sixteen-item scale adapted from Neal and Griffin [3]. The three measurement items of social influence were adapted from Venkatesh and Davis [22]. To assess shared decision making, a nine-item scale adapted from Baggs [49] and Dunn et al. [40] was used. A scale developed by Sorensen et al. [50] was adapted to six measurement items to assess preventive action. Safety satisfaction was assessed using six items adapted from Cheah et al. [7].

To ensure that the translated scale items reflected the same meaning as those in the original language, we interviewed several senior managers at hospital units and health care management scholars to evaluate the appropriateness of the items based on the characteristics of the medical industry. Their evaluations were used as the reference for design and modification of the pretest questionnaire. After semantically unclear words and sentences were modified and inappropriate items were deleted using this approach, we distributed 30 of the modified questionnaires in a hospital to conduct a pretest. The questionnaire was finalized after ensuring that all pretest participants understood the meaning of the questionnaire items. The final questionnaire was structured.

### 2.3. Statistical Tools and Methods

The data were analyzed using SPSS 18.0 (SPSS Inc., Chicago, IL, USA) and AMOS 18.0 (SPSS Inc., Chicago, IL, USA) software programs. Firstly, descriptive statistics were examined. Then, the two-stage approach recommended by Anderson and Gerbing [51] was used to evaluate the proposed model. Structural equation modeling (SEM) was used to examine the proposed model empirically. The goodness-of-fit statistics and commonly recommended threshold of SEM included: χ^2^/df < 8 (the ratio of chi-square to the degree of freedom), GFI > 0.8 (goodness-of-fit index), AGFI > 0.8 (adjusted goodness-of-fit index), RMSEA < 0.08 (root mean square error of approximation), RMR < 0.08 (root mean square residual), NFI > 0.9 (normed fit index), IFI > 0.9 (incremental index of fit), TLI > 0.9 (Tucker-Lewis index), and CFI > 0.9 (comparative fit index).

The common method variance (CMV) test, reliability, convergent validity, and discriminant validity were conducted to verify the adequacy of the measurement model for each construct. Harman’s single-factor test was used to identify the CMV. The procedure includes exploratory factor analysis (EFA) and confirmatory factor analysis (CFA). Secondly, the structural model of path relationships linking the six constructs was validated. Finally, the hypothesized model was tested and the results regarding standardized indirect and total effects were reported.

## 3. Results

### 3.1. Descriptive Characteristics

Among the participants, 19.7% (*n* = 92) were males and 80.3% (*n* = 376) were females. Most participants were married (49.4%, *n* = 231), followed by those who were single (48.3%, *n* = 226). In terms of educational levels, 84.6% (*n* = 396) reported having university education as their highest educational level, followed by graduate school (10.0%, *n* = 47). Most respondents were in the age group of 30–39 years (35.3%, *n* = 165), followed by 20–29 years (26.3%, *n* = 123). A majority of the respondents were nurses (38.9%), followed by medical technicians (27.4%). 201 of the respondents (42.9%) had 1–5 years of professional experience, and 72 had 6–10 years (15.4%).

### 3.2. Common Method Variance Analysis (CMV)

The results of the EFA conducted for 44 items measuring six constructs (perceived organizational support, safety climate, social influence, shared decision making, preventive action, and safety satisfaction) confirmed a six-factor structure. Using the criteria of eigenvalue of more than 1, six factors were extracted from 44 items, accounting for a total variance of 78.955%. Because 26.191% of total variance was explained by the first factor (less than 50%), the effect of common method bias should not be a problem (Table 1).

In addition, the results of the single latent factor CFA testing revealed that the factor loadings of 44 items were not all significant, which further verified the same findings of EFA.

### 3.3. Reliability and Validity

Table 2 reports Cronbach’s α, composite reliability (CR), and the average variance extracted (AVE) of all constructs. As the values of Cronbach’s α in Table 2 are all above Nunnally’s recommended threshold of 0.7 [52], suggesting internal consistency is considered adequate.

Convergent validity was validated by examining Cronbach’s α, CR, and AVE from the measures [53]. CR means the sum of a latent variable’s factor loadings relative to the sum of the factor loadings plus error variance. From Table 2, the Cronbach’s alpha of all constructs is well above the acceptability value 0.7 [52]. In addition, the composite reliability (CR) of all constructs ranges from 0.901 to 0.977. Also, the average variance extracted (AVE) of all constructs ranges from 0.664 to 0.874 [53]. Therefore, the findings indicate that measurements possess high convergent validity.

Table 4 shows the correlation coefficient values among the latent constructs with square roots of AVEs shown on diagonal elements. According to Fornell and Larcker [54], bivariate correlation coefficient of any construct with other constructs should be less than the square roots of the AVE of the construct. This is observed for all constructs in Table 3. Therefore, the discriminant validity of all constructs is acceptable.

### 3.4. Structural Equation Modeling

The data were analyzed using the AMOS software. Overall model fit, which refers to how well the overall fit is between the model and the data. The fit indices of structural equation modeling obtained for the proposed conceptual model revealed that χ^2^/df = 2.720 (*p* < 0.001), GFI = 0.83, AGFI = 0.81, RMSEA = 0.05, RMR = 0.04, NFI = 0.92, IFI = 0.95, TLI = 0.94, and CFI = 0.95. All fit indices were within the recommended threshold value, demonstrating a good overall model fit of the structural model to the data.

### 3.5. Hypotheses Testing

**Figure 2:**
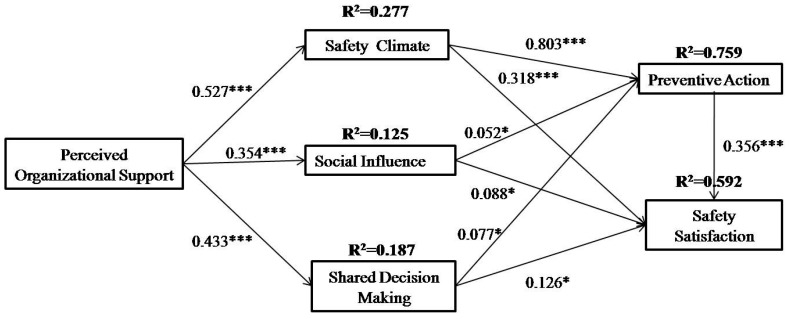
Final proposed model. *** *p* < 0.001; * *p* < 0.05.

The final structural model with the estimated standardized path coefficients and path significance among the constructs is presented in Figure 2 and Table 4. As hypothesized, all of the proposed hypotheses are supported. Safety climate (*β* = 0.803, *p* < 0.001), social influence (*β* = 0.052, *p* < 0.05), and shared decision making (*β* = 0.077, *p* < 0.05) jointly significantly affected preventive action, collectively accounting for 75.9% of the variance (R^2^ = 0.759) in preventive action. Supports were found for the following hypotheses: H1, H4, and H6. Safety climate (*β* = 0.318, *p* < 0.001), social influence (*β* = 0.088, *p* < 0.05), shared decision making (*β* = 0.126, *p* < 0.05), and preventive action (*β* = 0.356, *p* < 0.001) jointly significantly affected safety satisfaction, accounting for 35.3% of the variance in safety satisfaction. Therefore, H2, H3, H5, and H7 were all supported. 

According to H8, perceived organizational support was related positively to safety climate. This hypothesis was supported (*β* = 0.527, *p* < 0.001). Also, perceived organizational support explained 27.7% of the variance in safety climate. H9 examined the relationship between perceived organizational support and social influence. This hypothesis was also support (*β* = 0.354, *p* < 0.001). The 12.5% of the variance in social influence was explained by perceived organizational support. In addition, perceived organizational support (*β* = 0.433, *p* < 0.001) significantly affected shared decision making, accounting for 18.7% of the variance in shared decision making. Thus, H10 was supported. Table 5 summarizes the results for the hypotheses. 

Table 5 lists the standardized indirect and total effects in the proposed model. As shown in Table 5, safety climate exerted the strongest overall effect (0.803) on preventive action, followed by perceived organizational support (0.475). In addition, safety climate also exerted the strongest overall effect (0.604) on safety satisfaction, followed by perceived organizational support (0.422).

## 4. Discussion 

In terms of total effects on the results of preventive action and safety satisfaction, safety climate ranked first and directly, or indirectly, influenced safety satisfaction through preventive action. This showed the importance and relevance of safety climate. In other studies, safety climate was also an antecedent variable of crucial indicators of other safety performance factors such as occupational injuries. Therefore, management must not passively wait for an occupational injury to realize the importance of safety climate. Managers must constantly modify and improve the safety climate and encourage and reward safe behaviors and preventive actions. Moreover, they should avoid punishment and criticism of unsafe behavior; only in this way can proactive behavior occur to reduce occupational injuries and increase safety satisfaction [55]. For medical institutions, relevant institutional safety reporting mechanisms and protocols should be resorted to when a patient safety incident occurs. Subsequently, improvement strategies should be proposed, and managers should adopt a positive and proactive attitude toward discussing occupational safety concerns and incidents. 

Interventions aiming to strengthen a safety climate have mostly focused on increasing employees’ compliance with safety regulations. Feedback and incentives have been used to alter behaviors. However, Neal and Griffin suggested that managers should simultaneously consider, observe, and analyze the advantages and disadvantages of employee participation and compliance. Managers should examine the cause of a problem and determine whether its cause is a lack of knowledge or skills (for which education and training should be provided) or a lack of motivation (for which factors such as leadership style, atmosphere, work design, and personal attitude should be adjusted) [3]. Hofmann and Mark adopted a similar perspective and observed that overall, establishing a safety climate involves developing high-quality safety practices, encouraging employees to comply with these practices, and implementing effective learning when errors occur. Therefore, a comprehensive evaluation of safety climate should include not only the quality of safety practices but also the degree to which the social context encourages compliance with these practices. Additionally, approaches for effective and constructive response to errors should be included [6]. 

Research on social influence is rare in the domain of OSH. Nevertheless, social influence has been emphasized in the domain of health care because it involves patient safety. Studies have verified that social influence or social norms are key social factors affecting the safety behavior of medical staff toward patients [24]. In terms of creating organizational norms, organizations must promote occupational and patient safety, create safety operation standards, compile guidance, and establish norms for each department and unit, providing relevant education, training, and interventions where necessary. Additionally, hospitals can hold cross-division and cross-unit experience sharing events or workshops regarding common occupational injuries (e.g., needle stick injuries), create a social network on which successful cases and their methods of treatment can be communicated, and remind employees of procedures that can easily result in occupational injuries; all these activities have positive influences and can strengthen social influences. 

This study verified that shared decision making had positive effects on preventive action and safety satisfaction. To achieve successful results through shared decision making, all team members must communicate their viewpoint and knowledge, and their contributions must be understood and valued. Moreover, willingness to accept other team members’ perspectives and respecting and trusting them are crucial factors affecting the success of shared decision making. According to Towle and Godolphin [56], the realization of shared decision making requires clear definitions of the necessary knowledge, skills, and abilities. Therefore, implementing shared decision making requires systematic support, education plans, and an excellent atmosphere and culture. Only in this way can shared decision making become routine. 

In this study, perceived organizational support was identified as the antecedent variable of three variables: safety climate, social influence, and shared decision making. Therefore, perceived organizational support is crucial in terms of increasing the effectiveness of the three aforementioned factors. According to the perspective of reciprocity norms, when workers perceive that their organization supports them, values their contribution, and is concerned for their wellbeing, their obligation is triggered, and they exhibit prosocial behaviors. Accordingly, when high-level managers of an organization place greater emphasis on plans related to occupational safety, social influence, and shared decision making and provide the necessary resources, rewards, and assistance, employees take these plans for granted. Subsequently, the employees proactively participate in extrarole behaviors. As the agents of an organization, high-level managers can open communication channels and create an atmosphere of trust across units. 

Hospitals have highly complex structures and processes, in addition to having people as their service target, who are more important than products. To meet their social responsibility and manage their institution sustainably, hospitals must emphasize workplace health and safety while simultaneously providing high-quality service. Employees are the greatest assets of any institution, and only healthy employees can pay the greatest attention to the health and safety of patients. Hospital operations involve contact with biologically, chemically, and radioactively hazardous substances; thus, OSH are fundamental elements of the environment that cannot be ignored. Additionally, medical staff should be given the above incentives and strategies for engaging in preventive behaviors. 

Hospitals are places where the general public receives treatment for diseases and for health promotion. Therefore, medical staff in hospitals must set an excellent example by strictly practicing safe occupational behaviors. This forms a virtuous cycle of team norms and organizational climate and thus creates a high-quality medical environment, achieving the ultimate goal of protecting patients and improving the health of the general public. Moreover, hospital staff and managers must provide their employees with a healthy workplace. In this way, employees’ work satisfaction and organizational identification can be increased. Moreover, such actions can have a synergistic effect on employee safety and health and enable employees to enjoy their work, become health protection ambassadors, and plan a brighter future for the health of the general public. 

## 5. Conclusions

The present study explored and verified a causal structural framework of the outcomes of hospital staff’s perceptions of OSH. Structural equation modeling was used to verify the conceptual research framework proposed in this study. As predicted, all hypotheses in this study were supported. Additionally, the explained variance of the two outcome variables, preventive action and safety satisfaction, was high, and the goodness-of-fit of the model is well. The findings of the present study suggest the importance of joint consideration of perceived organizational support, safety climate, social influence, and shared decision making to improve the levels of prevention actions and safety satisfaction. Accordingly, the research framework of this study obtained excellent results and can serve as a basis and reference for future research.

## Figures and Tables

**Table 1:** Results of common method variance analysis.

Factor	Initial Eigenvalues		
	Total	% of Variance	Cumulative %
1	11.524	26.191	26.191
2	8.013	18.211	44.403
3	5.128	11.655	56.058
4	3.779	8.589	64.647
5	3.541	8.048	72.695
6	2.755	6.260	78.955

**Table 2:** Results of reliability and validity analyses.

Construct	Cronbach’s α	CR	AVE
Perceived Organization Support	0.917	0.918	0.737
Safety Climate	0.971	0.970	0.664
Social Influence	0.899	0.901	0.753
Shared Decision Making	0.969	0.969	0.776
Preventive Action	0.961	0.959	0.798
Safety Satisfaction	0.977	0.977	0.874

Notes: Cronbach’s α: internal consistency; CR: Composite Reliability; AVE: Average Variance Extracted.

**Table 3:** Results of discriminant validity analysis.

Construct	1	2	3	4	5	6
1. Perceived Organization Support	(0.90)					
2. Safety Climate	0.46	(0.82)				
3. Social Influence	0.36	0.51	(0.87)			
4. Shared Decision Making	0.39	0.70	0.50	(0.88)		
5. Preventive Action	0.47	0.80	0.50	0.63	(0.89)	
6. Safety Satisfaction	0.53	0.70	0.51	0.60	0.71	(0.94)

**Table 4:** Summary of hypotheses validated results.

Hypothesis	Path Coefficients	Results
H1 Safety climate → Preventive action	0.803 ***	Supported
H2 Safety climate → Safety satisfaction	0.318 ***	Supported
H3 Preventive action → Safety satisfaction	0.356 ***	Supported
H4 Social influence → Preventive action	0.052 *	Supported
H5 Social influence → Safety satisfaction	0.088 *	Supported
H6 Shared decision making → Preventive action	0.077 *	Supported
H7 Shared decision making → Safety satisfaction	0.126 *	Supported
H8 Perceived organizational support → Safety climate	0.527 ***	Supported
H9 Perceived organizational support → Social influence	0.354 ***	Supported
H10 Perceived organizational support → Shared decision making	0.433 ***	Supported

Notes: *** *p* < 0.001; * *p* < 0.05.

**Table 5:** Results of standardized indirect and total effects analysis.

Construct	Safety Climate	Social Influence	Shared Decision Making	Preventive Action	Safety Satisfaction
Perceived Organization Support	NA/0.527	NA/0.354	NA/0.433	0.475/0.475	0.422/0.422
Safety Climate	-	-	-	NA/0.803	0.286/0.604
Social Influence	-	-	-	NA/0.052	0.019/0.107
Shared Decision Making	-	-	-	NA/0.077	0.027/0.153
Preventive Action	-	-	-	-	NA/0.356
Safety Satisfaction	-	-	-	-	-

Notes: Numbers before the slash represent indirect effects, numbers after the slash represent total effects; NA means not applicable.
